# Transcutaneous Electrical Stimulation and Dysphagia Rehabilitation: A Narrative Review

**DOI:** 10.1155/2020/4865614

**Published:** 2020-05-11

**Authors:** Ali Barikroo

**Affiliations:** Swallowing Physiology and Rehabilitation Research Laboratory, Speech Pathology and Audiology Program, Kent State University, Kent, Ohio, USA

## Abstract

Transcutaneous electrical stimulation (TES) was introduced as a modality for dysphagia rehabilitation more than a decade ago. The underlying premise of this modality is improving the structural movements and enhancing neural activation based on stimulation-induced muscle contractions. However, divisive evidence exists regarding the effectiveness of this treatment modality. This manuscript reviews current evidence regarding the effects of transcutaneous electrical stimulation (TES) on clinical and physiological aspects of swallowing function. Furthermore, this narrative review delineates the knowledge gap in this area and recommends future research roadmap. This review gives a comprehensive picture regarding current knowledge of TES to practicing speech and language pathologists and interested researchers. It highlights the need for more robust studies in this area. It also encourages researchers to focus more on the physiologic studies to understand the physiologic underpinning behind this treatment modality.

## 1. Introduction

Transcutaneous electrical stimulation (TES) has been used for several decades in other rehabilitation fields for a variety of therapeutic aims, such as strengthening muscles, increasing joint range of motion, preventing muscle atrophy, reducing pain, increasing sensory awareness, and enhancing the healing process [1]. TES is a reasonably new treatment modality in swallowing therapy, having been introduced in 2002 [2]. TES is purported to strengthen weak swallowing muscles and improve swallowing function in patients with dysphagia. Many studies have been conducted to understand the impact of TES on different aspects of swallowing function. In the following, the effects of TES on the clinical and physiological aspects of swallowing are being reviewed.

### 1.1. Effects of TES on Clinical Aspect of Swallowing

The effects of TES on the clinical aspects of swallowing rehabilitation have been studied to a significant extent but with conflicting outcomes. For example, an early study [2] reported superior clinical benefits of TES over conventional swallowing therapy in patients with dysphagia secondary to stroke. However, no randomization was performed for participants. Furthermore, no standard protocol was used to evaluate patients' function during videofluoroscopy. In a retrospective cohort study, Blumenfeld et al. [3] found superior treatment benefits for dysphagia therapy with TES compared to traditional dysphagia therapy in individuals in a long-term acute care facility. Nevertheless, the findings of this study should be considered in light of the inherent limitations of a retrospective cohort design, such as a lack of control over confounding factors and investigators' bias. In a case-control study, Kushner et al. [4] compared the effect of TES combined with traditional dysphagia therapy and traditional dysphagia therapy alone following stroke, reporting significantly enhanced outcomes for the combined therapy approach. Again, the findings of this study should be interpreted in the context of its limitations, such as a nonrandomized procedure for selecting the patients, the unequal size of the case and control groups, and no masking for the therapist and/or evaluator regarding the participant's assigned group. However, these same findings were later replicated in studies with stronger research designs [5, 6]. Combining TES with other modalities has shown promising results in other neurologic populations as well. For example, Tang et al. [7] showed that combining TES with surface electromyography (sEMG) can improve swallowing function, nutritional status, and airway safety in patients with Alzheimer's disease. Finally, Chen et al. [8], in a meta-analysis of eight randomized and quasi-randomized controlled trials, reported that using TES combined with other swallowing therapy approaches is potentially more effective than using TES alone for treating patients with poststroke dysphagia. However, they stated a lack of evidence supporting TES apart as superior to swallowing therapy. Apart from the peripheral impact on improving the strength of swallowing muscles (i.e., peripheral contribution pathway), TES may also have the potential to treat dysphagia through enhancing CNS sensory input [9]. This resonated sensory input can increase neural activation in CNS circuits, which can facilitate motor unit recruitment (i.e., central contribution pathway) [10]. In this regard, a recent study has suggested that combining TES with bilateral repetitive transcranial magnetic stimulation (rTMS) can lead to higher cortical activation and better swallowing function in patients with stroke [11].

Contrary to these positive clinical outcomes for TES, some studies have reported equivocal or weaker clinical benefits for TES-based dysphagia treatment compared with traditional therapy. For example, two similar randomized clinical trials in patients with dysphagia secondary to Parkinson's disease [12, 13] reported no enhanced clinical benefit of TES when combined with traditional dysphagia therapy. Using a meta-analysis, Tan et al. [14] reported that TES was comparable to conventional dysphagia therapy following stroke. Some studies have compared the effect of TES on swallowing with other exercise-based programs. Guillén-Solà et al. [15] compared the impact of inspiratory/expiratory muscle strength training with TES, and traditional treatment on swallowing function. The findings of this study indicated that both groups A and B had comparable impacts on improving the swallowing safety. In a randomized clinical trial, Carnaby et al. [16] compared three treatment approaches in patients with dysphagia: traditional dysphagia therapy, McNeill Dysphagia Therapy Program (MDTP) with sham TES, and MDTP with TES. The results indicated that TES did not improve the effect of MDTP; instead, MDTP with TES showed fewer clinical benefits than MDTP with sham TES. In a double-blinded randomized clinical trial, Langmore et al. [17] compared two therapy approaches in patients with dysphagia following head and neck cancers: TES plus swallowing exercises versus sham TES plus swallowing exercises. This study demonstrated no added benefit of TES when combined with traditional swallowing exercises. Instead, based on the penetration aspiration scale, patients with sham TES experienced safer swallows than patients with TES.

In summary, a great deal of controversy exists in the literature regarding the clinical effectiveness of the existing TES protocols. While these studies vary significantly in quality and methodology, the majority of the studies reported that using the existing TES protocol alone might not have the desired clinical effects on the swallowing system. Alternatively, they described that combining the current TES protocol with other dysphagia rehabilitation techniques has induced better clinical outcomes.  Table 1 summarizes the published studies regarding the effect of TES on the clinical aspect of swallowing.

### 1.2. Effects of TES on Physiological Aspects of Swallowing

A modest amount of evidence exists regarding the physiological impact of TES on swallowing muscles. One group of studies focused on the immediate effect of TES on the kinematic aspect of swallowing. For example, Ludlow et al. [18] applied TES on both submental and infrahyoid areas in 11 patients with dysphagia, indicating an immediate lowering effect of TES selectively on the hyoid bone at rest. However, this study was limited only to a small sample of patients and was underpowered. In a larger study, Humbert et al. [19] studied the effect of 10 electrode placements on a hyolaryngeal excursion at rest and during swallowing in 29 healthy adults. In an extension to Ludlow et al.'s study, they reported not only a descending pattern for the hyoid bone both at rest and during swallowing but also for the larynx. This descending effect of TES has been referred to the contraction of hyolaryngeal depressor muscles. These muscles are larger and closer to the neck surface compared with the hyolaryngeal elevator muscles. As a result of this anatomy, stimulation of the infrahyoid area primarily impact hyolaryngeal depressor muscles, thereby pulling down the hyolaryngeal complex. In a similar study, Kim et al. [20] tested the effects of three different electrode placements on hyolaryngeal positions in 20 healthy adults and found similar descending patterns for both the hyoid and larynx at rest for all three different electrode placements. In another study, Lee et al. [21] compared the immediate effect of two electrode placements (both submental and infrahyoid regions versus submental placement alone) on hyolaryngeal position in 15 patients with dysphagia. The findings of this study were in line with the majority of previous studies indicating an immediate lowering effect of TES on the hyolaryngeal complex during stimulation of both submental and infrahyoid regions. Given the immediate descending effect of TES on hyolaryngeal excursion, Park et al. [22] used effortful swallow as an exercise paradigm against the resistance of lowered hyoid under TES in healthy adults. The results of this study demonstrated that superior hyoid excursion increased after two weeks. Nam et al. [23] later replicated this study paradigm in dysphagia patients with brain injury and reported similar findings. Beyond this demonstrated impact of existing TES protocol on swallowing kinematics, few studies have investigated the immediate impact of TES on other swallowing physiology measures. For example, using conventional manometry, Heck et al. [24] reported no immediate effect of TES on pharyngeal pressure in 27 healthy young adults. Nevertheless, they described delayed effects of TES that manifested as decreased hypopharyngeal pressure and increased upper esophageal sphincter (UES) relaxation, which continued for up to one hour after stimulation. Given the lack of immediate pharyngeal pressure response (i.e., peripheral effect), the authors have assumed the observed delayed effect is centrally driven by the brainstem (i.e., central effect). In a forward step, some studies investigated the effect of different TES parameters on swallowing function. In this regard, Berretin-Felix et al. [25] reported differential effects of sensory versus motor TES amplitudes on lingual-palatal and pharyngeal pressure in healthy young versus older adults. The results of this study demonstrated that high-amplitude TES reduced anterior and posterior lingua-palatal peak pressures but increased hypopharyngeal peak pressures for both age groups. Low-amplitude TES increased the base of tongue (BOT) peak pressures in older adults but reduced BOT peak pressures in younger adults. Similarly, in a secondary analysis of archived pressure data, Barikroo et al. [26] reported differential effects of TES amplitudes on lingual-palatal and pharyngeal pressure timing across age groups. Specifically, they reported no immediate effect of high-amplitude TES on lingual-palatal and pharyngeal pressure timing during swallowing for both healthy young and older adults. Conversely, they reported faster pharyngeal pressure during swallowing for older adults following low-amplitude TES. Recently, new studies have focused on optimizing the TES protocol for swallowing. For example, Barikroo et al. [27] showed that using short pulse duration (300 *μ*s) compare with long pulse duration (700 *μ*s) could further increase the maximum amplitude tolerance (MAT) without increasing the perceived discomfort level. The authors concluded that increasing MAT after using short pulse duration potentially stimulates deeper swallowing muscles. In a follow-up study, the same group of authors tested the effect of submental TES with varying pulse durations on lingual-palatal pressure measures during swallowing. They reported that using short pulse duration significantly decreased lingual-palatal pressure compared with long pulse duration [28]. This finding was also replicated in another study. Specifically, Takahashi et al. [29] investigated the effect of laryngeal TES with short pulse duration (200 *μ*s) on swallowing performance. They reported decreased tongue pressure and hyoid elevation during swallowing. These studies may suggest that using TES with short pulse duration may help us in triggering swallowing muscles that are buried in deeper tissue layers such as genioglossus, hyoglossus, and thyrohyoid. Pulse frequency is another TES parameter that impacts the quality of muscle contractions and likely swallowing physiology. The pulse frequency is associated with modulating the firing rate of motor unit recruitments and the strength of muscle contractions [30]. The majority of existing TES protocols use an 80 Hz frequency to stimulate these muscles. This frequency potentially fatigues the swallowing muscles, which should typically be stimulated at 30 Hz [31]. Furthermore, some studies suggest that using a pulse frequency at the range of 1-120 Hz induces greater discomfort when compared with kilohertz frequencies. Moreover, kilohertz frequencies induce less skin impedance and can penetrate deeper through the tissues [32]. In this regard, Jungheim et al. [33] utilized high-resolution manometry to compare the effect of two TES protocols with 2700 Hz and 100 Hz on swallowing pressure. The findings of this study indicated that the TES protocol with kilohertz frequency increased the base of tongue retraction. However, no significant effect of frequency was observed on the upper esophageal sphincter opening. Additional studies are needed to understand the effect of varying TES frequency on fatigue and swallowing function. One of the other TES parameters that need to be explored is the electrical stimulation waveform. Current TES waveforms for swallow rehabilitation apply square waves [34–38]. However, literature relating to other parts of the body indicates that a sine waveform has deeper penetration through human tissue [37–45], which can be beneficial if we need to target deep swallowing muscles. It is now clear that a one-size-fits-all approach for TES-based dysphagia rehabilitation is simplistic. Therefore, the TES parameters should be tailored based on the pathophysiology of swallowing. More studies are needed to understand the effect of other TES parameters on swallowing physiology.

In summary, the majority of studies recommend that applying TES on submental and infrahyoid regions lowers the tongue and hyolaryngeal complex. Furthermore, some studies have indicated that this TES-induced hyolaryngeal descending effect can be used as a resistive exercise paradigm to improve the tongue strength and hyolaryngeal excursion during swallowing. Beyond the kinematic effect, recent evidence suggests that various TES amplitude levels may have a distinctive modulation impact on the swallowing physiology across age groups. Specifically, older adults benefit more from certain TES amplitudes than younger adults in some measured physiologic activities and more significant deterioration in other aspects of swallowing physiology. Furthermore, using short pulse duration may increase maximum amplitude tolerance that can subsequently increase the depth of electrical current penetration. Moreover, the lack of information exists regarding the effect of pulse frequency on swallowing physiology.  Table 2 summarizes the published literature regarding the effect of TES on the physiological aspect of swallowing.

## 2. Conclusions and Future Research Directions

The gist of conducted clinical trials suggests that TES works best as an adjunct modality when it is combined with other rehabilitation techniques. In addition, it seems that TES-based dysphagia rehabilitation has been more successful in a certain group of etiologies (i.e., stroke) than the others (i.e., head and neck cancer, progressive neurological disorders). However, this message has not been consistent across different studies. Part of this controversy may relate to the weak methodological designs for many of the performed studies such as small sample size, inadequate patient selection criteria, lack of control group, lack of control over confounding variables, short-term follow-up periods, and use of nonstandardized tests for evaluating outcome measures. Thus, having robust multicenter clinical trials can help us to have a clear picture regarding the effectiveness of this modality alone or in combination with other treatment modalities across different etiologies. Furthermore, it is essential to incorporate the results of physiological studies into clinical trials. That is what I call a physiological-based TES rehabilitation approach. Based on this approach, the underlying physiological issues of swallowing should be identified first (i.e., lower hyolaryngeal excursion, decreased base of tongue pressure, or delayed laryngeal vestibule closure), and the TES protocol should be adjusted in a way to address that specific pathophysiology. As a result, more studies are required to understand the effect of TES on different aspects of swallowing physiology. Many clinical studies were conducted with preset TES parameters (i.e., electrode size and placement, waveform, amplitude, frequency, and pulse duration) with no information regarding the rationale behind their decision. It is now clear that a one-size-fits-all approach for TES-based dysphagia rehabilitation is misdirected. Each TES parameter has the potential to change the quality of swallowing muscle contractions, which may impact on the physiology of swallowing distinctively and ultimately alter the treatment outcomes. Beyond the effect of TES parameters, biopsychological characteristics of each patient, such as subcutaneous adipose tissue thickness, pain sensitivity, and fear of pain may modify the effects of TES on swallowing function. As a result, more studies are needed to understand the combined effect of different TES parameters and biopsychological factors on swallowing physiology and dysphagia rehabilitation. Finally, additional studies are required to understand the impact of TES on neural activation and neural plasticity in patients with varying etiology.

## Figures and Tables

**Table 1:** Summaries of studies regarding the effects of transcutaneous electrical stimulation (TES) on the clinical aspect of swallowing.

Study	Study design	Sample size	Dysphagia etiology	Type of intervention	Outcome measures	Key findings
Freed et al. [2]	Case-control study	11	Stroke	TES vs. TT	Swallow function score	Swallowing function was improved in both groups. The score change was greater in TES vs. TT group.

Blumenfeld et al. [3]	Retrospective cohort study	80 (40 patients and 40 controls)	Mixed	TES vs. TT	Swallow severity scale	Patients who underwent TES demonstrated better swallowing function.

Kushner et al. [4]	Case-control study	92 (46 patients and 46 controls)	Stroke	TES+TT vs. TT	FOIS	Both TES+TT and TT improved swallowing functions. Swallowing function was greater when TES was combined with TT compared with TT alone.

Lee et al. [5]	RCT	57 (31 patients and 26 controls)	Stroke	TES+TT vs. TT	FOIS	Both TES+TT and TT improved swallowing functions. Swallowing function was greater when TES was combined with TT compared with TT alone.

Sun et al. [6]	Case-series	29	Stroke	TES+FEES+TT	FOIS	Combined dysphagia rehabilitation (TES+FEES+TT) improved swallowing function.

Tang et al. [7]	Retrospective cohort study	103 (53 patients, 50 control)	Alzheimer's disease	TES+sEMG vs. TT	Water swallow test. MNA aspiration pneumonia	Swallowing function, nutritional status, and airway safety were better in the experimental group

Ortega1 et al. [9]	RCT	38 (19 patients and 19 controls)	Aging	Sensory TES vs. capsaicin	EAT-10 PAS VFSS	Both therapies improved the safety of swallow and oropharyngeal swallow response.

Zhang1 et al. [11]	RCT	64 (16 TES+sham rTMS vs. 16 TES+ipsilateral rTMS vs. 16 TES+contralateral rTMS vs. 16 TES+bilateral rTMS)	Stroke	TES+sham rTMS vs. TES+ipsilateral rTMS vs. TES+contralateral rTMS vs. TES+bilateral rTMS)	Motor evoked potential, standardized swallowing assessment	Bi-rTMS/TES produced higher cortical activation and better swallowing function.

Baijens et al. [12]	RCT	90 (30 TT, 30 motor TES, and 30 sensory TES)	Parkinson's disease	Motor TES+TT vs. Sensory TES+TT vs. TT	Visuoperceptual ordinal variables in FEES and VFSS	Both TES groups had no significant impacts on swallowing function

Heijens et al. [13]	RCT	85 (26 TT, 27 motor TES, and 30 sensory TES)	Parkinson's disease	Motor TES vs. sensory TES vs. TT	FOIS SWAL-QOL MDADI DSS	DSS was significantly improved after treatment for all groups. Limited improvements on the SWAL-QOL and the MDADI for all groups. No significant differences were observed between groups.

Guillén-Solà et al. [15]	RCT	62 (21 TT, 21 TT+IEMT, and 20 sham IEMT+ TES)	Stroke	TT vs. TT+IEMT vs. sham IEMT+TES	PAS maximal inspiratory and expiratory pressures	Maximal respiratory pressures were mostly improved in group two (TT+IEMT). Swallowing security signs were improved in both groups two (TT+IEMT) and three (sham IEMT+ TES). No differences in PAS or respiratory complications were detected among three groups.

Carnaby et al. [16]	RCT	53 (17 TT, 18 TES+ MDTP, 18 sham TES+MDTP)	Stroke	TT vs. TES+MDTP vs. sham TES+MDTP	MASA FOIS	TES+MDTP had poor outcome compared with sham TES +MDTP

Langmore et al. [17]	RCT	127 (91 patients and 36 controls)	HNC	TES+TT vs. sham TES+TT	PAS OPSE VFSS PSS HNCI	TES+TT group had worse PAS scores compared with the control group. Nutrition and quality of life were improved for both groups. No other significant changes compared with baseline for both groups.

TES: transcutaneous electrical stimulation; RCT: randomized controlled trial; TT: traditional treatment; FOIS: functional oral intake; FEES: fiberoptic endoscopic examination of swallowing; SWAL-QOL: Swallowing Quality of Life Questionnaire; MDADI: MD Anderson dysphagia inventory; DSS: dysphagia severity scale; VFSS: videofluoroscopic swallowing study; MDTP: McNeill Dysphagia Therapy Program; PAS: penetration aspiration scale; OPSE: oropharyngeal swallow efficiency; PSS: performance status scale; HNC: head neck cancer; HNCI: head and neck cancer inventory; EAT-10: Eating Assessment Tool 10; IEMT: inspiratory/expiratory muscle training; sEMG: surface electromyography; MNA: mini nutritional assessment; rTMS: repetitive transcranial magnetic stimulation.

**Table 2:** Summaries of studies regarding the effect of transcutaneous electrical stimulation (TES) on the physiological aspect of swallowing.

Study	Study design	Sample size	Swallowing function	Electrode placement	Outcome measures	Key findings
Ludlow et al. [18]	Cross-sectional	11	Dysphagia	Submental and throat	Hyoid and laryngeal positions at rest	Increasing hyoid excursion at rest
Humbert et al. [19]	Cross-sectional	29	Healthy	10 electrode placements	Hyolaryngeal excursion at rest and during swallowing	Decreasing hyoid and laryngeal excursions at rest and during swallowing
Kim et al. [20]	Cross-sectional	20	Dysphagia	Three electrode placements	Hyolaryngeal excursion at rest	Decreasing hyoid and laryngeal excursion at rest
Lee et al. [21]	Cross-sectional	15	Dysphagia	Submental and throat vs. submental	Hyolaryngeal excursion at rest	Submental/throat regions: decreasing hyolaryngeal excursion Submental: increasing anterior excursion of the hyolaryngeal structures
Park et al. [22]	Case-series	16	Healthy	Throat	Hyolaryngeal excursion during swallowing	Increasing superior hyoid excursion
Nam et al. [23]	Case-control	50	Dysphagia	Submental and throat vs. submental	Hyolaryngeal excursion during swallowing	Submental: increasing hyoid excursion during swallowing; submental/throat regions: increasing laryngeal excursion
Heck et al. [24]	Cross-sectional	20	Healthy	Submental	Hypopharyngeal and UES PP	Immediate effect: no TES effect on PP Delayed effect: decreasing hypopharyngeal PP and increasing UES relaxation
Berretin-Felix et al. [25]	Cross-sectional	34 (20 young, 14 older adults)	Healthy	Submental and throat	Lingual-palatal, BOT, hypopharyngeal, UES PP	Motor TES: decreasing anterior tongue pressure in older adults, increasing hypopharyngeal pressure for both age groups Sensory TES: increasing BOT pressure in older adults, decreasing BOT pressure in younger adults
Barikroo et al. [26]	Cross-sectional	34 (20 young, 14 older adults)	Healthy	Submental and throat	Lingual-palatal, BOT, hypopharyngeal, UES pressure timing	Motor TES: no effect Sensory TES: faster PP for older adults
Barikroo et al. [28]	Cross-sectional	30 older adults	Healthy	Submental	Lingual-palatal pressure	Short pulse duration decreased lingual palatal peak pressure compared with the long pulse duration
Takahashi et al. [29]	Cross-sectional	18 young	Healthy	Throat	Lingual-palatal pressure and hyoid excursion	Decreasing tongue pressure during stimulation and was significantly lower after stimulation Decreasing the hyoid position at rest Increasing the hyoid position both at rest and during the maximum excursion during stimulation
Jungheim et al. [33]	Cross-sectional	29	Healthy	Submental	Velopharynx, BOT, and UES pressure	Midfrequency stimulation: increasing BOT pressure No impact on UES Low-frequency pressure: no significant changes in any parameters examined

TES: transcutaneous electrical stimulation; UES: upper esophageal sphincter; BOT: base of tongue; PP: pharyngeal pressure.
